# Update on Perineuronal Net Staining With *Wisteria floribunda* Agglutinin (WFA)

**DOI:** 10.3389/fnint.2022.851988

**Published:** 2022-04-01

**Authors:** Wolfgang Härtig, Anton Meinicke, Dominik Michalski, Stefan Schob, Carsten Jäger

**Affiliations:** ^1^Paul Flechsig Institute for Brain Research, University of Leipzig, Leipzig, Germany; ^2^Department of Neurology, University of Leipzig, Leipzig, Germany; ^3^Department of Neuroradiology, Clinic and Policlinic of Radiology, University Hospital Halle, Halle (Saale), Germany; ^4^Department of Neurophysics, Max Planck Institute for Human Cognitive and Brain Sciences, Leipzig, Germany

**Keywords:** perineuronal net, extracellular matrix (ECM), WFA, aggrecan, CSPG, cerebral ischemia

## Abstract

As chemically specialized forms of the extracellular matrix in the central nervous system, polyanionic perineuronal nets (PNs) contain diverse constituents, including chondroitin sulfate proteoglycans (CSPGs), hyaluronic acid, and tenascins. They are detectable by various histological approaches such as colloidal iron binding and immunohistochemical staining to reveal, for instance, the CSPGs aggrecan, neurocan, phosphacan, and versican. Moreover, biotin, peroxidase, or fluorescein conjugates of the lectins *Vicia villosa* agglutinin and soybean agglutinin enable the visualization of PNs. At present, the *N*-acetylgalactosamine-binding *Wisteria floribunda* agglutinin (WFA) is the most widely applied marker for PNs. Therefore, this article is largely focused on methodological aspects of WFA staining. Notably, fluorescent WFA labeling allows, after its conversion into electron-dense adducts, electron microscopic analyses. Furthermore, the usefulness of WFA conjugates for the oftentimes neglected *in vivo* and *in vitro* labeling of PNs is emphasized. Subsequently, we discuss impaired WFA-staining sites after long-lasting experiments *in vitro*, especially in autoptic brain samples with long *postmortem* delay and partial enzymatic degradation, while immunolabeling of aggrecan and CSPG link proteins under such conditions has proven more robust. In some hippocampal regions from perfusion-fixed mice, more PNs are aggrecan immunoreactive than WFA positive, whereas the retrosplenial cortex displays many WFA-binding PNs devoid of visible aggrecan immunoreactivity. Additional multiple fluorescence labeling exemplarily revealed in ischemic tissue diminished staining of WFA-binding sites and aquaporin 4 and concomitantly upregulated immunolabeling of neurofilament, light chains, and collagen IV. Finally, we briefly discuss possible future staining approaches based on nanobodies to facilitate novel technologies revealing details of net morphology.

## Introduction

Lattice-like coatings around neurons were first revealed by Camillo Golgi and his Italian colleagues at the end of the 19th century as summarized by Celio et al. (1998). In parallel, such structures resembling honey webs were described by Ramón y Cajal and his coworkers as reported by Brauer et al. (1982) when they introduced the designation “perineuronal nets” (PNs). Brückner et al. (1993) gave a first overview of PNs surrounding certain highly active neurons and emphasized the complexity of these polyanionic structures containing mainly chondroitin sulfate proteoglycans (CSPGs) such as aggrecan as well as hyaluronic acid and tenascins.

Functions of PNs as chemically specialized forms of the extracellular matrix (ECM) in the brain are still not fully elucidated but include their role in limiting the plasticity during ontogenesis (Hockfield et al., 1990) and critical period regulation (Reh et al., 2020), as cation exchangers (Härtig et al., 1999) and diffusion barriers with ion sorting properties (Morawski et al., 2015). Very recently, PNs were shown to stabilize the grid network that supports navigation and spatial memory (Christensen et al., 2021). The growing interest in PNs as forms of the ECM is reflected by numerous reviews focused, for instance, on PNs and perinodal ECM regulating neuronal functions (Fawcett et al., 2019) and the control of plasticity during CNS maturation (Sorg et al., 2016; Carulli and Verhaagen, 2021). Disturbed or degraded PNs were observed after experimentally induced focal ischemia (Härtig et al., 2017) and affected PNs contribute to schizophrenia (Pantazopoulos and Berretta, 2016). The steadily increased interest for PNs under pathological conditions is documented by several reviews (e.g., Testa et al., 2019; Wen et al., 2018; Reichelt et al., 2019).

Polyanionic PNs were initially detected by histological methods, such as multifaceted Golgi techniques (Brauer et al., 1982; Celio et al., 1998), while later being frequently visualized by colloidal iron binding (Brückner et al., 1993), Gömöris ammoniacal silver, and Ehrlich’s methylene blue (Murakami et al., 1999). Among the subsequently developed antibody-based techniques to detect net components, the immunolabeling of aggrecan, the predominantly found CSPG in PNs, is widely used. Various aggrecan glycoforms contribute to the molecular heterogeneity of PNs (Matthews et al., 2002; Miyata et al., 2018).

Starting in the 1980s, the *N*-acetylgalactosamine-binding lectins such as *Vicia villosa* agglutinin (VVA) and soybean agglutinin (SBA) were established as additional net markers by applying their conjugates with peroxidase (Nakagawa et al., 1986), biotin (Kosaka and Heizmann, 1989), and fluorescein (Mulligan et al., 1992).

Subsequently, biotinylated *Wisteria floribunda* agglutinin (WFA) was introduced as a robust and selective marker for PNs predominantly surrounding GABAergic, parvalbumin-containing neurons (Härtig et al., 1999). Recently, WFA was shown to bind both non-reducing terminal and internal *N*-acetylgalactosamine residues to recognize heparin and to specifically interact with non-sulfated tetrasaccharides of the O–O type (Nadanaka et al., 2020). WFA has been applied to reveal PNs in numerous mammalian species and even in amphibians (Edwards et al., 2021), chicken (Morawski et al., 2009) and seasonally altered in canary birds (Cornez et al., 2020).

This short article is focused on the technical aspects of lectin histochemistry with WFA. Thereby, newly established triple fluorescence labeling is applied to reveal differences between the staining patterns of WFA-binding sites and aggrecan immunoreactivity (ir) around parvalbumin-containing neurons. To consider disease-related effects regarding PNs, analyses include not only naïve tissues but also ischemia-affected brain regions. Moreover, aspects of conventional as well as not commonly used approaches based on WFA conjugates are discussed.

## Experimental Procedures

In general, experiments were performed following the ethical guidelines as given by the European Union Directive 2010/63/EU and were approved by the locally responsible authority (Regierungspräsidium Leipzig). Three-month-old mice underwent permanent middle cerebral artery occlusion (pMCAO) according to Hawkes et al. (2013) and were sacrificed by perfusion with phosphate-buffered 4% paraformaldehyde after a period of 24 h. After removal from the skulls, the whole brains were post-fixed with the same fixative overnight and equilibrated with 30% phosphate-buffered sucrose. Series of 30-μm-thick forebrain sections were then cut with a freezing microtome (Leica SM 2000R, Leica Biosystems, Wetzlar, Germany). Prior to use, all sections were stored at 4°C in sealed vials filled with 0.1 M Tris-buffered saline, pH 7.4 (TBS) with sodium azide as an additive.

All staining experiments were started by extensive washing with TBS followed by the blocking of potential non-specific binding sites by treatment for 1 h with 5% normal donkey serum in TBS containing 0.3% Triton X-100 (NDS-TBS-T).

For the detection of net components and parvalbumin-ir in ischemia-free control regions, the sections were incubated for 20 h with a mixture consisting of biotinylated WFA (B-1355-2; Vector Labs, Burlingame, CA, United States; 15 μg/ml NDS-TBS-T), rabbit-anti-aggrecan (AB1031; Merck Millipore, Billerica, MA, United States; 1:200), and guinea pig-anti-parvalbumin (195004; Synaptic Systems, Göttingen, Germany; 1:300). The sections were then rinsed with TBS and processed for 1 h with a cocktail containing Cy2-tagged streptavidin, Cy3-donkey-anti-rabbit IgG, and Cy5-donkey-anti-guinea pig IgG [all from Jackson ImmunoResearch, West Grove, PA, United States; 20 μg/ml TBS containing 2% bovine serum albumin (TBS-BSA)].

Next, ischemia-affected sections underwent the concomitant staining of biotinylated WFA and goat-anti-collagen IV as described earlier (Härtig et al., 2017) and combined either with the immunodetection of neurofilament, light chain (NF-L) as reported by Härtig et al. (2016) or aquaporin 4 (AQP4) according to Hawkes et al. (2013). In brief, sections were first incubated for 20 h with mixtures of biotinylated WFA (15 μg/ml NDS-TBS-T), goat-anti-collagen IV (AB769; Merck Millipore; 1:100), and either rabbit-anti-NF-L (171002; Synaptic Systems; 1:200) or guinea pig-anti-AQP4 (429004; Synaptic Systems; 1:200). The markers were then reacted for 1 h with a mixture consisting of Cy2-streptavidin, AlexaFluor647-donkey-anti-goat IgG, and either Cy3-donkey-anti-rabbit IgG or Cy3-donkey-anti-guinea pig IgG (all from Jackson ImmunoResearch; 20 μg/ml TBS-BSA).

After extensive rinses with TBS, all sections were briefly washed with distilled water, mounted onto fluorescence-free glass slides, air-dried, and coverslipped with Entellan in toluene (Merck, Darmstadt, Germany).

In histological control experiments, the omission of WFA and antibodies resulted in the expected absence of any cellular and ECM staining.

Micrographs were made with a microscope Biorevo BZ-9000 (Keyence, Neu-Isenburg, Germany) and processed with PowerPoint for Mac (Office 365, version 2021; Microsoft Corp., Redmond, WA, United States). The contrast and brightness of micrographs were slightly adjusted while avoiding the creation or deletion of fluorescence signals.

## Results and Discussion

The Toolbox (on page 4) summarizes the 30-year-long experience with WFA as a marker for PNs. While some listed points deserve no additional comments, it should be emphasized that most of them are in line with recently published general protocols for lectin histochemistry from Rebelo et al. (2021). However, it should be accentuated that preferable WFA concentrations strongly differ between sensitive immunoperoxidase staining (0.5–1 μg/ml) and detection with fluorescent streptavidin conjugates (10–20 μg/ml).

An applicable, but rarely used option is the labeling with fluorescein-tagged WFA (FITC-WFA) which can be enhanced by Cy2-anti-fluorescein or converted by using anti-fluorescein-horseradish peroxidase (HRP) into a light microscopic visible, electron-dense DAB adduct in an analogous manner as reported for FITC-albumin (Michalski et al., 2010).

Notably, PNs are well detectable even in living mammalian tissues. For example, Brückner et al. (1996a) injected biotinylated WFA into rat brains and revealed PNs 6 days later in fixed tissue sections by a streptavidin/biotin technique. Moreover, these authors demonstrated that Cy3-tagged WFA was applicable in living tissues, and pre-labeling of PNs with Cy3-WFA in living tissues has been used to facilitate subsequent electrophysiological analyses within roughly 1 h of labeling (Hoppenrath et al., 2016).

**FIGURE 1:**
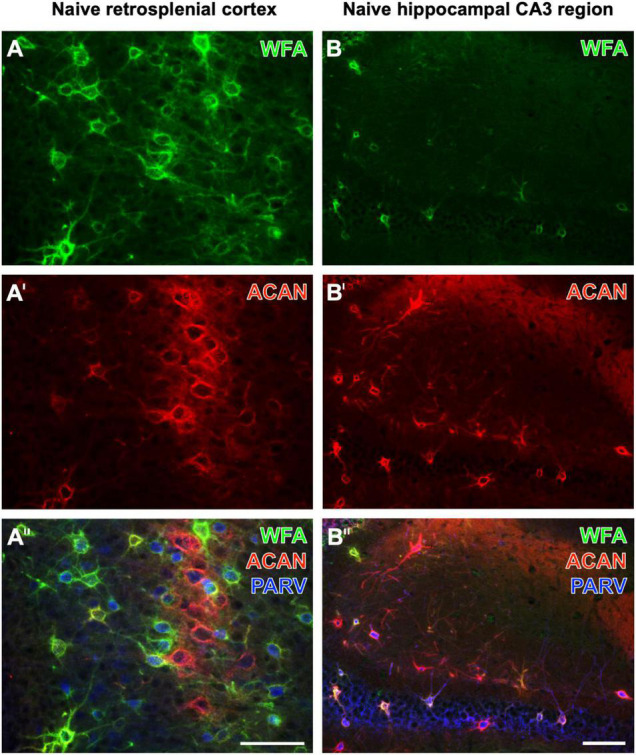
Triple fluorescence labeling of the net markers WFA and aggrecan (ACAN) combined with the immunodetection of parvalbumin (PARV) exemplarily shown for the retrosplenial cortex **(A–A′′)** and the hippocampal CA3 region **(B–B′′)** in non-affected control tissue from young adult mice. In **(A)**, the lectin-histochemical Cy2 staining of WFA reveals numerous PNs in the retrosplenial cortex, which also displays considerable ACAN-ir in **(A′)**. The overlay in **(A′′)** elucidates several net-like structures with lectin-binding sites but devoid of visible ACAN-ir, frequently around PARV-containing neurons. In parallel and as expected, many PNs are positive for both markers. WFA-positive structures in the CA3 region appear sparsely scattered **(B)**, whereas more ECM structures exhibit ACAN-ir **(B′)**. The overlay elucidates that net components with ACAN-ir outweigh those with WFA binding and are associated with PARV-immunoreactive structures. Scale bars **(A′′)** (also valid for **A,A′**), 75 μm, **(B′′)** (also valid for **B,B′**), 75 μm.

While Yamada and Jinno (2017) found that the vast majority of aggrecan-immunoreactive PNs were colocalized with WFA-binding sites, they counted a substantial population of PNs devoid of WFA labeling in the stratum oriens. Ueno and coworkers had described numerous WFA-positive, but aggrecan-immunonegative PNs in the mouse cerebral cortex (Ueno et al., 2018). Aggrecan immunolabeling can be enhanced by the pretreatment of fixed tissues with chondroitinase ABC (Carulli et al., 2010; Madinier et al., 2014), which would concomitantly prevent the WFA staining (Köppe et al., 1997). Focusing on naïve brain regions, this study shows the retrosplenial cortex as a mouse brain region displaying several PNs with strong WFA staining, but devoid of aggrecan-ir, at least under the applied imaging conditions (Figures 1A–A′′). While WFA-stainable *N*-acetylgalactosamine moieties are known as parts of aggrecans and contribute to the complexity of aggrecan forms (Matthews et al., 2002; Miyata et al., 2018), it might be possible that such moieties are also coupled with other components of nets with no or low amounts of aggrecan. Having in mind the lower molecular weight of biotinylated WFA (less than 70 kDa) in comparison with rabbit-anti-aggrecan IgG molecules (ca. 150 kDa), a different penetration depth of both markers might be considered. Conversely, we found in the CA3 region more aggrecan-immunoreactive PNs than WFA-positive PNs (see Figures 1B–B′′). This finding is in line, for instance, with quantitative data on the molecular heterogeneity of aggrecan-based PNs in the mouse hippocampus (Yamada and Jinno, 2017).

**FIGURE 2:**
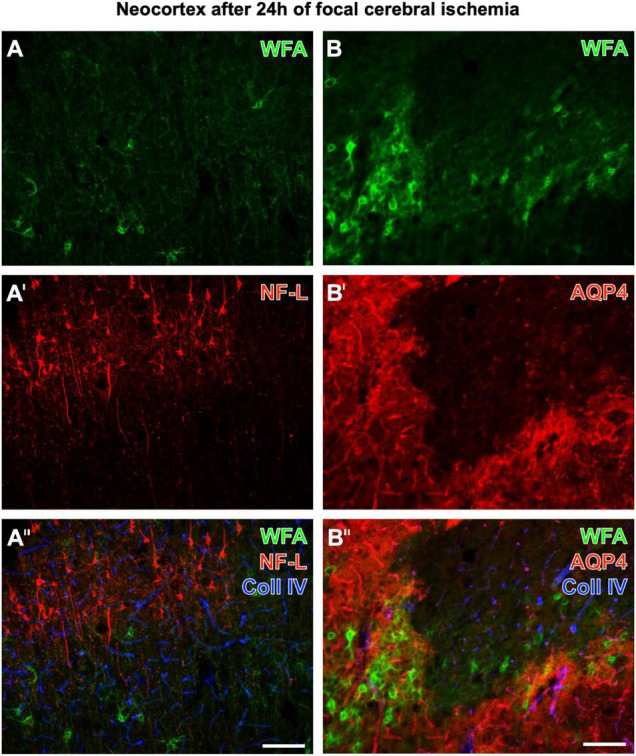
Concomitant fluorescence staining of WFA and collagen IV (Coll IV) in the ischemia-affected neocortex 24 h after ischemia onset combined with the immunolabeling of NF-L **(A–A′′)** or AQP4 **(B–B′′’)**. The green fluorescent WFA staining of PNs is diminished or erased in the ischemic tissue, which becomes obvious for the presented border zone **(A,B)**. In parallel, affected tissue displays a heavily upregulated NF-L-ir in the upper cortical layers and frequently in pyramidal cells with long apical dendrites **(A′)**. In the first overlay **(A′′)**, ischemic areas are additionally pronounced by strongly enhanced collagen (Coll) IV-ir color-coded in blue. Next, panel **(B′)** elucidates that in the infarcted region AQP4, as a marker for astrocytic endfeet visualizing vessels under physiological conditions, is drastically altered (as visible in the lower right part of the micrograph) or even abolished. The second overlay also shows that Coll IV-ir is upregulated in areas devoid of PNs and AQP4-ir, and also in tissue with damaged PNs and diffuse AQP4-ir. Scale bars **(A′′)** (also valid for **A,A′**), 100 μm, **(B′′)** (also valid for **B,B′**), 100 μm.

Figure 2 displays representative examples of altered, diminished, and erased PNs in neurological disease. Twenty-four hours after ischemia onset induced by pMCAO in mice, neocortical WFA-stained PNs are strongly affected in ischemic areas, which concomitantly show upregulated collagen IV-ir (in line with Härtig et al., 2017). Figures 2A–A′′ show additional staining of strongly enhanced NF-L-ir in the ischemic tissue, whereas Figures 2B–B′′ are completed by the simultaneous detection of a further highly sensitive marker, AQP4. In ischemia-affected areas, AQP4-ir was either abolished or diffusely distributed.

**FIGURE 3:**
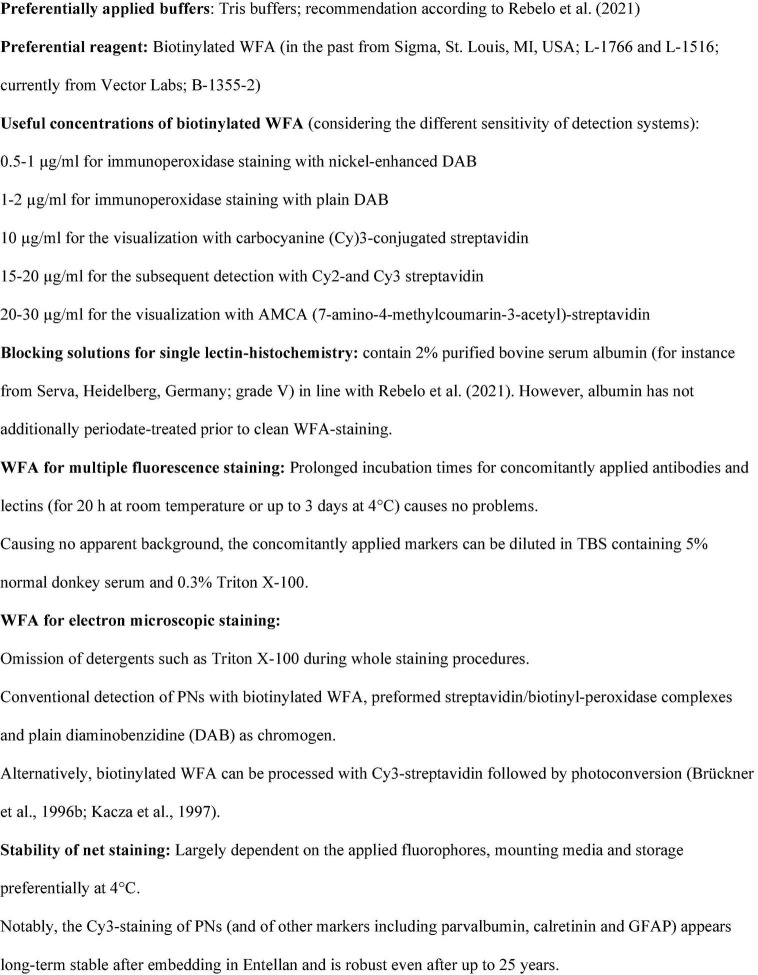
**Toolbox:** Prerequisites for successful WFA-staining.

Notably, the WFA staining is compromised and often impossible to detect PNs in autoptic tissues and even after long-lasting electrophysiological approaches with living tissues as reported by Wegner et al. (2003). As demonstrated by Morawski et al. (2012), after prolonged *postmortem* delay, WFA binding declined while aggrecan-ir remained largely unaffected. Furthermore, WFA staining might also be diminished in autoptic tissues by *premortem* history and pathology and, in general, by long-term storage in conventional buffers at 4–6°C for several years. *N*-Acetylgalactosamine moieties are obviously more susceptible to enzymatic degradation than the protein components of aggrecan and the link proteins that are targeted by CSPG-cleaving enzymes (Levy et al., 2015; Rossier et al., 2015).

Recently provided detailed protocols for the visualization of PNs (Souter and Kwok, 2020) and automated analyses of net staining intensities (Slaker et al., 2016; Venturino and Siegert, 2021) might be extended to reveal a larger fraction of all nets. Thereby, mixtures of biotinylated WFA and rabbit-anti-aggrecan could be visualized by the same color (e.g., Cy2) or by differing fluorophores. Considering brevican as the main component of perisynaptic axonal coats (Morawski et al., 2012) and the importance of tenascins, brevican, and neurocan for the formation of complete net structures (Brückner et al., 2000; Gottschling et al., 2019), such approaches might be extended by the concomitant detection of the aforementioned net constituents.

Future studies of PNs should comprise the use of nanobodies with lower molecular weight and easier penetration of tissues than conventional antibodies. Nanobodies recognizing aggrecan and WFA (fragments) could be applied as mixtures or as conjugates.

Novel staining technologies might support the super-resolution imaging of maturing PNs (Sigal et al., 2019) and sophisticated techniques for the fine structure analyses of PNs, for instance, in animal models of schizophrenia (Kaushik et al., 2021) and in focal cerebral ischemia and mild hypoperfusion (Dzyubenko et al., 2018).

## Data Availability Statement

The raw data supporting the conclusions of this article will be made available by the authors, upon reasonable request.

## Ethics Statement

The animal study was reviewed and approved by the Regierungspräsidium Leipzig.

## Author Contributions

WH wrote the manuscript and performed the staining experiments. CJ, SS, DM, and AM revised the manuscript. AM and DM generated the figures. SS, CJ, and WH contributed to essential data in the Toolbox (Figure 3). All authors contributed to the article and approved the submitted version.

## Conflict of Interest

The authors declare that the research was conducted in the absence of any commercial or financial relationships that could be construed as a potential conflict of interest.

## Publisher’s Note

All claims expressed in this article are solely those of the authors and do not necessarily represent those of their affiliated organizations, or those of the publisher, the editors and the reviewers. Any product that may be evaluated in this article, or claim that may be made by its manufacturer, is not guaranteed or endorsed by the publisher.
